# Spontaneous splenic rupture associated with Q fever and portal hypertension: A case report

**DOI:** 10.3389/fmed.2025.1527701

**Published:** 2025-02-11

**Authors:** Cecil Mustafiz, Deloshaan Subhaharan, Daniel Chorley, Tariq Masood

**Affiliations:** ^1^Department of Digestive Health, Gold Coast University Hospital, Gold Coast, QLD, Australia; ^2^School of Medicine and Dentistry, Griffith University, Gold Coast, QLD, Australia

**Keywords:** splenic rupture, portal hypertension, cirrhosis, Q fever, hepatitis C, hepatology, splenectomy, infectious diseases

## Abstract

Spontaneous splenic rupture (SSR) is an exceptionally rare and life-threatening condition, with its pathophysiology remaining poorly understood. This report describes the first documented case of SSR in a patient with Q fever infection and underlying liver cirrhosis with portal hypertension. The patient was a man in his late 30 s who presented with severe abdominal pain, vomiting and hypovolemic shock who required emergency splenectomy due to unstable splenic hemorrhage. Post-operatively, a diagnosis of Q fever was confirmed through serological testing. To date, there has only been six cases describing splenic rupture precipitated by Q fever, and none in the context of concurrent portal hypertension. Conversely, portal hypertension is an independent risk factor for splenic complications including splenomegaly and hypersplenism. This case underlines the critical need to consider rare etiologies, offers valuable insights into the pathogenesis of SSR, and emphasizes the importance of early recognition and multidisciplinary management. Moreover, a proposed algorithm for the diagnosis and management of SSR has been included for clinicians who face similar complex presentations.

## 1 Introduction

Spontaneous splenic rupture (SSR) is a remarkably uncommon condition which carries high mortality (1). Among infectious etiologies of SSR, Q fever is rare with only six documented cases (2–9). It is caused by the gram-negative bacterium *Coxiella burnetti* and spread through direct and airborne contact with animals including cattle, sheep and goats. Individuals who reside with livestock are particularly susceptible (3). While a majority of patients remain asymptomatic in acute infection (60%), symptoms usually manifest after an incubation period of 2–6 weeks and typically include fever, cough, myalgia and arthralgia. Atypical pneumonia, hepatitis and hepatosplenomegaly have also been reported. A positive diagnosis is confirmed with serum PCR and two positive serological tests which measure immunoglobulin levels 2–3 weeks apart (10). Acute Q fever is detectable on PCR testing up to 2 weeks after illness onset, while a rise in IgM to Phase II antigens usually occurs 10–14 days after onset. First-line treatment for acute Q fever includes a 14-day course of 100 mg oral doxycycline twice daily (11–13).

Aside from infective etiologies of SSR, other risk factors include hematologic, neoplastic and chronic inflammatory conditions. Among chronic conditions, portal hypertension is a significant risk factor for splenic complications including splenomegaly and hypersplenism, potentially predisposing individuals to increased susceptibility of splenic rupture (14, 15). While portal hypertension and infectious processes like Q fever have been studied in isolation, the combined effect of these distinctive risk factors creates a unique pathophysiological environment, which magnifies the risk of SSR. By reviewing previously reported cases of Q fever-associated SSR and their outcomes, this report aims to expand understanding of SSR’s diverse etiologies and guide future clinical decision-making.

## 2 Case description

A Caucasian man in his late 30 s was brought into emergency with a 1-day onset of severe generalized abdominal pain and vomiting. He was well in the days prior and denied any fevers, night sweats, joint pain, rashes, diarrhea, dysuria, or cough. His background was significant for untreated hepatitis C and alcoholic Child Pugh B liver cirrhosis with established portal hypertension. He did not have any significant family or psychosocial history. He required multiple hospital admissions over the past year due to decompensated liver disease with ascites. The patient’s regular medications included pantoprazole and thiamine. He consumed 16 standard alcoholic drinks weekly and smoked cigarettes and marijuana daily without use of any other illicit substances.

On initial presentation, vital signs were within normal ranges without evidence of hepatic encephalopathy. Physical examination revealed a distended abdomen with generalized tenderness with no signs of peritonism or ascites. Despite analgesia, he reported ongoing abdominal pain, and shortly after, the patient collapsed when mobilizing to the bathroom. Vital signs were now consistent with shock (blood pressure 78/49 mmHg), with repeat abdominal examinations revealing global peritonism.

### 2.1 Investigations

**FIGURE 1:**
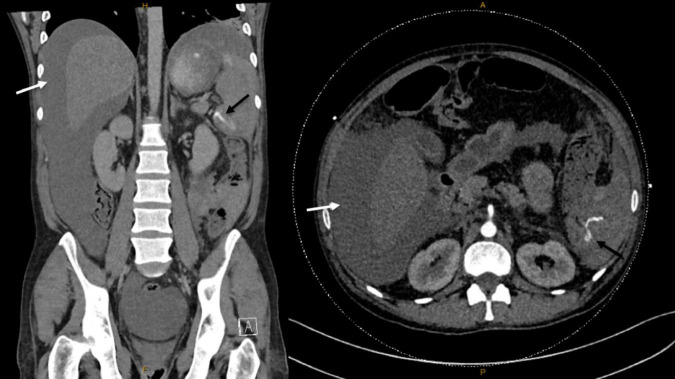
CT angiography (delayed venous and arterial phase) of the abdomen and pelvis. White arrow: significant intraperitoneal fluid accumulation due to hemoperitoneum. Black arrow: hyperdense foci due to acute bleeding from the inferior pole of the spleen with a surrounding subscapular splenic hematoma consistent with splenic rupture.

Laboratory results revealed a significant drop in hemoglobin from 104 to 69 g/L, indicating acute blood loss. Additionally, there was a marked elevation in white blood cell count (21.2 × 10^9^/L) and C-reactive protein (19 mg/L), suggesting an inflammatory/infectious process. Other laboratory tests, including liver function tests, coagulation studies, serum creatinine, and electrolytes, remained stable compared to previous baseline values. Chest X-ray and urine culture were unremarkable. Diagnostic abdominal paracentesis drained blood-stained ascitic fluid; however, both microscopy and culture were negative for spontaneous bacterial peritonitis. Venous blood gas analysis indicated metabolic acidosis (pH 7.31) and a high lactate level of 4.9 mmol/L, further supporting the presence of systemic shock. An urgent abdominal CT angiography was conducted, which revealed a large volume hemoperitoneum and active bleeding from the inferior pole of the spleen, consistent with spontaneous splenic rupture (Figure 1). Given the findings and the patient’s critical hemodynamic instability, a decision was made to proceed with urgent splenectomy.

The choice of splenectomy, rather than other interventions such as conservative management or embolization, was based on thorough clinical evaluation and interdisciplinary collaboration between the surgical, hepatology, interventional radiology and intensive care teams. All teams agreed that an emergency splenectomy was the best treatment modality which could be offered, over other management strategies. Splenectomy was performed successfully without any complications and the patient was closely monitored in intensive care unit post-operatively.

Histopathological analysis of the spleen revealed expansion of red pulp and diminished white pulp tissue, consistent with the patient’s known portal hypertension. Macroscopically, there was evidence of mild splenomegaly (spleen size was 14.5 cm). One week post-surgery, the patient developed a fever of 39.3°C, along with fatigue, malaise and a dry cough. Repeat laboratory tests demonstrated elevated white blood cell count (29.3 × 10^9^/L) and C-reactive protein (115 mg/L). Chest X-ray revealed hazy consolidation in the left mid and lower zones and the right perihilar region. Extended respiratory viral serology, sputum cultures and blood cultures were all negative. However, a key finding was a positive Q fever quantitative PCR test, along with a reactive Phase II IgM on enzyme immunoassay. Phase II IgG was negative. A repeat Phase II IgM remained positive 2 weeks later, confirming acute Q fever infection.

**FIGURE 2:**
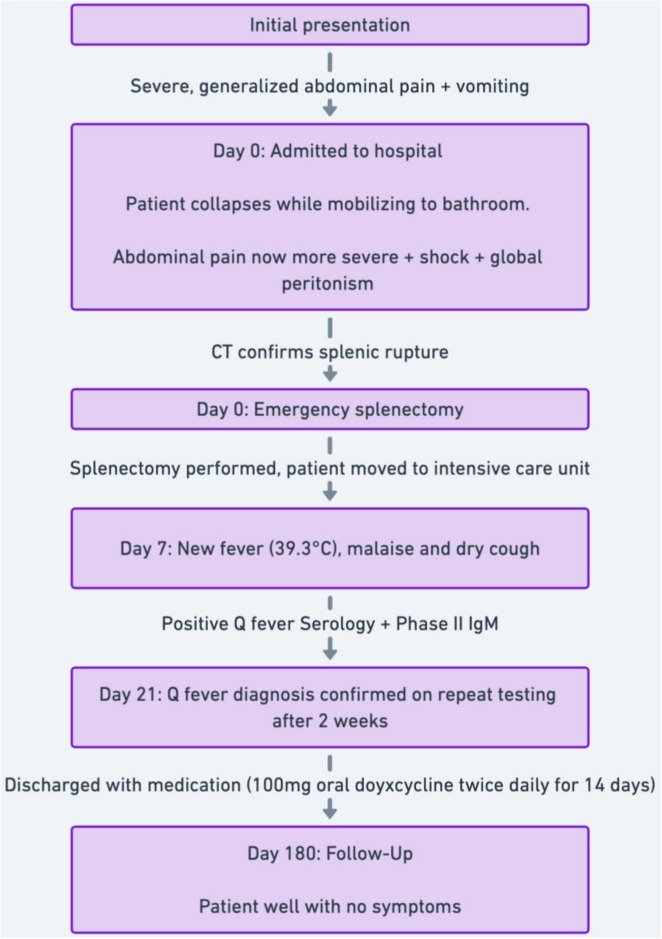
Clinical case timeline.

A summarized timeline of events has also been provided (Figure 2).

### 2.2 Outcome and follow-up

The patient was discharged with a 2-week course of oral doxycycline 100 mg twice daily and long-term amoxicillin for infection prophylaxis post-splenectomy. He was prescribed antivirals for his chronic hepatitis C and educated on the importance of ongoing vaccinations after splenectomy. At 6-month follow-up, the patient remained well, with no complications post-splenectomy, eradicated hepatitis C and alcohol abstinence.

## 3 Discussion

SSR is an exceedingly rare condition. It is estimated less than 0.5% of all splenic ruptures occur in the absence of trauma (16). A systematic review of 845 patients identified the most common etiologies, which included neoplastic (30.3%), infectious (27.3%), inflammatory (20%), drug-related (9.2%), mechanical (7%) and idiopathic (7%) causes. Another systematic review of 613 patients similarly found infectious and neoplastic causes were most common (17). Other risk factors for SSR include splenomegaly, age over 40 years, portal hypertension and delayed diagnosis or treatment, which are associated with poorer outcome (1, 14, 15, 18). Regardless of etiology, SSR has a high mortality of up to 20% (19).

Among infective causes, Q fever is extremely rare with only six documented cases (4–9). In all six cases, patients presented to hospital with splenic rupture following a duration of 2–14 days of Q fever symptoms including high fevers, malaise, arthralgia, myalgia and headache (4–9). Clinical or radiological evidence of atypical pneumonia was present in three cases (4, 6, 7). In a majority of reports, intraabdominal bleeding was suspected after sudden onset of abdominal pain and development of hypovolemic shock (4–9). Common laboratory findings included low hemoglobin, thrombocytopenia and elevated transaminases (5–9). In five out of six reports, treatment included emergency splenectomy, while one case was managed conservatively. In all six cases, antibiotic therapy with a course of doxycycline was provided, and all patients recovered well long-term (4–9).

There are several similarities between the above and our case. Clinical presentation, diagnosis and management were comparable with similar long-term positive outcomes. Nevertheless, a key distinction in our case was the development of Q fever symptomology 1 week after surgery. Our patient had a positive Q fever serology and an elevated Phase II IgM antigen 7 days after surgery, suggesting that the infection was in the incubation and early clinical phase at time of splenic rupture. In contrast to previous cases, our case is unique given the patient had two ultimate etiologies for SSR; hypersplenism secondary to the patient’s known portal hypertension, which was further compounded by acute Q fever infection.

The relationship between portal hypertension and SSR has been explored in various clinical contexts, highlighting distinct pathophysiological mechanisms including increased splenic congestion, vascular remodeling, and fragility of the splenic parenchyma (20). Portal hypertension leads to venous outflow obstruction and pooling of blood within the spleen, resulting in marked splenic congestion and hypersplenism. This engorgement increases intrasplenic pressure and tension, predisposing the spleen to rupture, even in the absence of trauma (21–23). Histopathological findings from multiple studies, consistently describe expanded red pulp, reduced white pulp and congestive changes in splenic parenchyma which was also found in our case (14, 15). In portal hypertension, the spleen becomes more vulnerable to mechanical forces, such as those exerted by abdominal musculature or diaphragmatic contractions, which can initiate capsular disruption and subsequent rupture (20, 24–29). Reticular endothelial hyperplasia and vascular remodeling secondary to portal hypertension can lead to vascular occlusion or infarction, which over time, increases the likelihood of splenic rupture (14, 20, 24–28). Infective processes, such as acute Q fever, can synergistically exacerbate the effects of portal hypertension (30). The systemic inflammatory response associated with infections, induces cytokine-mediated endothelial injury, further destabilizing splenic vasculature. In our case, this dual insult, created a unique milieu, which heightened the risk of SSR (14, 15, 21–29). Further exploration of the relationship between portal hypertension and SSR is critical to further understand the pathophysiology which may be best performed in animal models initially.

**FIGURE 3:**
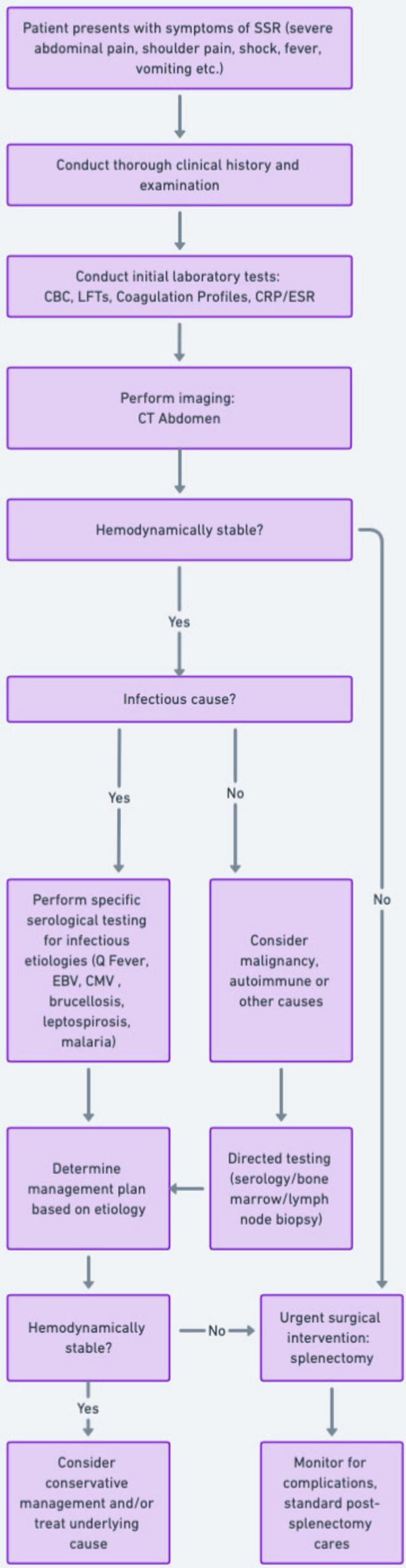
Flowchart: a guide for the diagnosis and management of spontaneous splenic rupture.

A focused systematic approach to SSR is essential to reduce morbidity and mortality. A simple algorithm is included (Figure 3). The diagnostic approach should begin with a thorough clinical history and physical examination to identify potential risk factors such as recent trauma, liver or splenic disease, and zoonotic or viral exposures (4–15). Patients with SSR frequently present with acute abdominal pain, often severe, and sometimes radiating to the left shoulder along with fever, vomiting, hypotension, and tachycardia (31–34). Laboratory investigations are vital for initial evaluation. These should include a complete blood count, liver function tests, coagulation profiles and inflammatory markers (14). Radiological imaging is critical, with computed tomography (CT) being the gold standard imaging modality. Bedside ultrasound could be useful to identify free intraperitoneal fluid (35, 36). Further investigations should be guided by suspicion of underlying etiology including neoplastic, infectious or inflammatory causes (37–41). Management strategies for SSR must be tailored to the patient’s hemodynamic status and underlying etiology. Unstable patients typically require emergency splenectomy, as recommended by the 2017 Word Society of Emergency Surgery guidelines, to control bleeding and prevent further hemodynamic compromise (42). Stable patients may be managed conservatively depending on the etiology and severity of splenic injury (43). Conservative management has demonstrated an 80% success rate in cases induced by infectious causes (1). Long-term management includes post-splenectomy care, where patients should receive pneumococcal vaccination to mitigate risks of infection along with amoxicillin prophlyaxis (44).

## 4 Conclusion

We have reported a unique case of SSR in the context of portal hypertension compounded by Q fever infection. It underlines the critical interplay of these distinct risk factors, which together created a unique pathophysiological environment for splenic injury. The case establishes the importance of interdisciplinary collaboration in recognizing rare but life-threatening conditions, facilitating timely diagnosis, and implementing effective management strategies. Moreover, a simple algorithm to guide clinicians to manage similar complex presentations is important.

## Data Availability

The original contributions presented in this study are included in this article/supplementary material, further inquiries can be directed to the corresponding author.
